# Patterns of illness disclosure among Indian slum dwellers: a qualitative study

**DOI:** 10.1186/s12914-018-0142-x

**Published:** 2018-01-16

**Authors:** Moumita Das, Federica Angeli, Anja J. S. M. Krumeich, Onno C. P. van Schayck

**Affiliations:** 1 School for Public Health and Primary Care, Maastricht University, Maastricht, the Netherlands; 2 Institute for Social and Economic Change (ISEC), Bangalore, India; 3 Department of Organization Studies, School of Social and Behavioral Sciences, Tilburg University, Tilburg, the Netherlands; 4 Department of Health Ethics and Society, School for Public Health and Primary Care, Maastricht University, Maastricht, the Netherlands; 5 Department of General Practice, School for Public Health and Primary Care, Maastricht University, Maastricht, the Netherlands

**Keywords:** Delay, Disclosure, Expressing illness, Gender, Informing illness, Slum dwellers

## Abstract

**Background:**

Slum dwellers display specific traits when it comes to disclosing their illnesses to professionals. The resulting actions lead to poor health-seeking behaviour and underutilisation of existing formal health facilities. The ways that slum people use to communicate their feelings about illness, the type of confidants that they choose, and the supportive and unsupportive social and cultural interactions to which they are exposed have not yet been studied in the Indian context, which constitutes an important knowledge gap for Indian policymakers and practitioners alike. To that end, this study examines the patterns of illness disclosure in Indian slums and the underpinning factors which shape the slum dwellers’ disclosing attitude.

**Methods:**

In-depth, semi-structured interviews were conducted among 105 men and 113 women who experienced illness in the year prior to the study period. Respondents were selected from four urban slums in two Indian cities, Bangalore and Kolkata.

**Results:**

Findings indicate that women have more confidants at different social levels, while men have a limited network of disclosures which is culturally and socially mediated. Gender role limitations, exclusion from peer groups and unsupportive local situations are the major cause of disclosure delay or non-disclosure among men, while the main concerns for women are a lack of proper knowledge about illness, unsupportive responses received from other people on certain occasions, the fear of social stigma, material loss and the burden of the local situation. Prompt sharing of illness among men is linked with prevention intention and coping with biological problems, whereas factors determining disclosure for women relate to ensuring emotional and instrumental safety, preventing collateral damage of illness, and preventing and managing biological complications.

**Conclusions:**

The findings reveal that patterns of disclosure are not determined by the acknowledgment of illness but largely depend on the interplay between individual agency, disclosure consequences and the socio cultural environment. The results of this study can contribute significantly to mitigating the pivotal knowledge gap between health policymakers, practitioners and patients, leading to the formulation of policies that maximise the utilisation of health facilities in slums.

## Background

In India, numerous healthcare policies aimed at improving the health conditions of the slum population have been adopted and implemented over the past decades. Nonetheless, the poor health status of the urban slum population has consistently been reported [1–3]. One of the factors that have been highlighted in previous studies on the poor health conditions of the slum population is their limited utilisation of healthcare resources [4–6]. Such underutilisation of care facilities often occurs because slum dwellers do not always disclose illnesses to and seek help from professionals [7], which leads to inadequate usage of healthcare.

In many societies, health problems are primarily handled within familial and social dimensions [8–10]. For instance, decisions such as when to seek aid and whom to consult take place in the lay arena based on the lay understanding and treating of illnesses [11]. Decision-making, however, not only involves the time of seeking and choosing care. Decisions also pertain to the way that illnesses are disclosed, in terms of which illness is revealed when, how and to whom [12–16]. The disclosure of illness is determined to a large extent by the favourable or unfavourable circumstances that exist within a socio cultural context [17–19]. Discussing illness with others is an important phase in health-seeking behaviour, as it is during this stage that a clear plan of action as to what should be done next (whether to keep it to oneself, see a specific doctor, go to a clinic, start self-medicating or wait for further symptoms to develop) is determined. For this reason, exploring how slum dwellers disclose illnesses and translate them into behaviours (deciding either to seek or to avoid help) could help policymakers in designing tailor-made measures to improve slum dwellers’ health status. Insight into illness-reporting patterns as well as into the type of confidants that slum dwellers choose is crucial to raise awareness of the available clinical and alternative healthcare treatments, which often remain underutilised to the detriment of slum dwellers’ health and wellbeing.

There is a great deal of literature in the field of chronic and mental illness [20–22] as well as sociocultural context [23, 24] on the extent to which people express their illness to others and on the way that society and culture in local settings influence disclosure patterns. However, very few comprehensive studies exist on how people deal with illnesses [25]. To our knowledge, no studies have considered the disclosure pattern of people residing in the slums of India with their unique socio cultural context [26].

The present study addresses this gap by investigating how slum dwellers in India disclose their illnesses. We particularly focused on the disclosure pattern before slum dwellers actually start their treatment process. Assuming that slum dwellers’ future plan of action completely relies on the lay discussion phase, it is crucial to understand the disclosure pattern during that stage in order to improve estimates of future healthcare demands and patterns of healthcare utilisation. To this end, this study specifically aims to uncover the many facets of lay decision-making before future action is taken and the reasons underpinning illness-expressing behaviour among Indian urban slum dwellers. The research questions can be formulated as follows:

- To whom and to what extent do slum dwellers decide to disclose their illnesses?
- What are the reasons not to disclose illness?
- What are the reasons underpinning a delay in disclosing illness?
- What are the reasons underpinning prompt disclosure of illness?

## Methods

### Study site

The present study was conducted in four urban slums of India equally selected from two cities, Kolkata in east India and Bangalore in south India. The slums were purposively selected on the basis of intra- and inter-geographical variation, age of the slum, religious plurality, variation in living conditions and the presence of different healthcare systems. The characteristics of the study areas, the Motijheel slum and SahidSmriti Colony of Kolkata as well as the Nakkle-Bande slum and UllaluUpanagar of Bangalore are given in Table 1.

**Table 1:** Characteristics of the Slums Studied

	Kolkata		Bangalore	
Name of the slum	Motijheel Slum (Core urban slum)	SahidSmriti Colony (Peri-urban slum)	Nakkle-Bande (Core urban slum)	UllaluUpanagar (Peri-urban slum)
Number of households	6000	2570	650	1500
Location	Highly congested slum	Sprawling slum surrounded by marshy land	Partly congested	Lots of space and barren land
Age of the slum (in years)	75	25	40	15
Origin of the population	Sub-urban Kolkata, Bihar, Jharkhand, Uttar Pradesh, Gujarat	Sub-urban Kolkata, Bangladesh, urban Kolkata	Sub-urban Karnataka, Tamil Nadu, AndhraPradesh	Displaced people from Bangalore city, rural Karnataka, Tamil Nadu, Andhra Pradesh
Social groups	Scheduled Caste and General	Scheduled Caste, Scheduled Tribe and General	Scheduled Caste, Scheduled Tribe and General	Scheduled Caste and General
Religion	Hindus (25%), Muslims (70%), Christians (5%)	Hindus (65%), Muslims (25%), Christians (10%)	Hindus (70%), Muslims (20%), Christians (10%)	Hindus (65%), Muslims (25%), Christians (10%)
Streets and roads	Maze-like alleys, paved	Simple streets, non-web and paved;bystreets, unpaved	Zigzagging, paved and non-spacious streets	Quite spacious, partly unpaved and partly paved
Type of houses	Pucca^a^ and semi-puccahouses	Kuccha,^b^ semi-pucca^c^and pucca houses	Pucca houses with three storeys	Kuccha, semi-pucca and pucca houses
Water supply	Thirtyto forty households with common pipedwater connection supplied by municipality	Hand pumps, ponds, few private tap connections and some public water taps	Private piped connectionsas well as public water connections for five families per street	Public water collection taps and few private piped connections; unplanned setup of all the water pipes, mostly located within the drains and sometimes with leakage, allowing the waste water to enter the pipes
Drainage	Both open and covered drainage	Unplanned and unsystematic open drainage within the slum	Underground drainage (in some places unplanned and unsystematic)	No proper drainage systems; waste water runs in both corners of the street
Toilet facilities	Approx. 40 households forone common toilet	Individual toilets	Individual toilets and two government-supplied community pay-and-use toilets	Few individual toilets and a public toilet run by a private organisation
Healthcare infrastructure	Two public hospitals, three private hospitals, two anganwadis, four medical clinics, six paramedical clinics, thirteen homeopathy clinics, two ayurveda clinics, one primary health centre, three midwives	One public and one private hospital, one anganwadi, two medical clinics, four paramedical clinics, seven homeopathy clinics, four ayurveda clinics, four midwives, thirteen traditional medicine men	Three general hospitals, two anganwadis, four nursing homes, seven maternity hospitals, two super-speciality hospitals, one cardiac and one orthopaedic hospital, twenty-sevenprivate clinics, one traditional medicine man, two ayurveda and four homeopathy clinics	One primary health centre, two anganwadis, nearest public hospital fifteen kilometres away, ten medical clinics, three midwives

Source: Author’s own calculations

^a^These houses are made for permanence and are built of substantial materials such as stone, brick, cement, concrete, iron, timber, and so on

^b^These crude houses are made on a temporary basis with wood, mud, straw and dry leaves

^c^These houses cannot be classified as either a permanentor a temporary structure. They have fixed walls made of solid materials, but their roof is made of the materials used in temporary houses

### Study participants

This study is part of a larger qualitative enquiry^1^ investigating health beliefs and practices among urban slum dwellers, which included 245 participants equally selected from both slums, of whom 129 males and 116 females. The socioeconomic profile of all the participants within the two study areas are shown in Table 2.

**Table 2:** Socio-Economic Profile of the Study Participants

Kolkata					Bangalore			
Motijheel (*n* = 69)			SahidSmriti (*n* = 66)		Nakkle-Bande (*n* = 48)		UllaluUpanagar (*n* = 62)	
	Men (*n* = 33)	Women (*n* = 36)	Men (*n* = 34)	Women (*n* = 32)	Men (*n* = 29)	Women (*n* = 19)	Men (*n* = 33)	Women (*n* = 29)
Age								
Undertwenties	4	3	3	3	2	3	3	4
Twenties	6	14	6	13	8	6	8	10
Thirties	10	9	12	7	12	5	12	8
Forties	7	6	10	6	4	4	6	4
Fifties	4	3	2	2	2	1	2	1
Sixties	2	1	1	1	1	0	2	2
Place of origin								
Rural	1	10	24	27	9	9	9	8
Within city	15	17	5	2	5	3	4	10
Displaced	6	0	0	0	4	0	16	9
Outside state	8	9	0	0	11	7	4	2
Outside country	3	0	5	3	0	0	0	0
Linguistic groups								
Hindi/Urdu	15	17	4	7	8	10	12	12
Bengali	18	19	30	25	0	0	0	0
Kannada	0	0	0	0	12	3	10	8
Tamil	0	0	0	0	5	3	6	7
Telugu	0	0	0	0	4	3	5	2
Family types								
Nuclear	18	10	6	8	23	17	20	24
Joint	15	26	28	24	6	2	13	5
Occupation								
Fully employed	19	16	16	1	15	7	19	6
Contractual	9	7	11	0	12	9	12	17
Unemployed	5	13	7	31	2	3	2	6
Monthly income (INR)								
0–2000	6	4	14	11	0	0	1	0
2001–4000	17	19	12	15	11	8	12	12
4001–6000	7	10	6	5	12	8	13	14
6000 and over	3	3	2	1	6	3	7	3

Source: Author’s own calculations

From this sample, 218 participants (105 men and 113 women) reported to the researcher to have experienced illness during the 12 months prior to the interview. Their subsequent responses towards illness gave rise to three different patterns of disclosure, to wit prompt disclosure, delayed disclosure and no disclosure at all.^2^ These participants were purposively selected for this study and were subsequently interviewed to investigate their behaviour in relation to illness disclosure. During the pilot study, we realised that a six-month recall period was too limited to collect extensive data on reporting behaviour. We attained data saturation after two to three interviews, since many participants reported not having experiencing any major or minor health incident in the past 6 months. As a result, a one-year recall period was used for both minor and chronic illnesses. A study by Kjellsson et al. [27] also indicates that using a recall period of 1 year is preferable to scaling up a recall period of one, three or 6 months. According to Sudman et al. [28], respondents are aware of the risks when being questioned on the recent past and prefer to disclose a more distant yet noteworthy event. As such an approach might influence their responses, it has been suggested as a way to reduce reporting errors. For multiple minor illnesses that occurred within a period of 1 year prior to the interview, the most recent case was considered for the study. The minimum age of the participants eligible for the study was fixed at 16 years, as it is assumed that the onset of puberty establishes self-awareness about one’s body and health.

### Data collection

In Kolkata, data on the health-seeking behaviour of slum dwellers were collected during August and October 2012. The main researcher personally conducted the interviews due to her familiarity with the local language (Bengali). All the respondents were told the purpose of the study, their part in the research, and the time and energy that they had to provide, as well as the possibility of pain, discomfort and stress that they might experience during the interview. Participation of the respondents was voluntary. Women were interviewed in their homes, while men were interviewed during their leisure period or sometimes in their workplace near their homes. The researcher audio-recorded the interviews and took notes. Each interview lasted for about 30 to 40 min. The Bangalore field study took place from July to December 2011. In-depth interviews were carried out in a conversational style with a semi-structured interview schedule for data collection. A female field investigator collected the field data under the guidance of the main researcher, as the local language (Kannada) was unfamiliar to the latter. Prior to the start of the fieldwork, the investigator received 1 week of guidance from the researcher to understand the scope and objective of the study, and to conduct mock interviews and pilot interviews. All interviews were either conducted in a separate room at the participants’ homes or in a separate spot at their workplace away from other people, in order to safeguard the privacy of the participants.

A semi-structured interview schedule was constructed according to the basic framework of Arthur Kleinman’s Explanatory Model of illness (EM) [29, 30]. The questions were of an open-ended nature in keeping with the emic perspective of this study, which aims to gather an in-depth understanding of the sociocultural context, respondents’ perceptions and self-constructed meanings [31–34]. The researcher had already established a rapport with the participants through frequent visits in the field, participation in their day-to-day activities, and the first session of interviews where we explored and discussed their perception of health. To understand the reporting behaviour of the participants, the interview schedule encompassed questions on perceptions of the concept of illness, the threshold for recognising illness, aetiology of illness, types of confidants and the type or nature of illness (minor, chronic, communicable, reproductive and sexually related ailments). Whether or not this information was shared underpins the causes of disclosure attitudes and the timing of disclosure. Probing questions were used for clarification if more information was needed to explore issues raised by the respondents or to confirm the validity of their answers through cross-checks. Prior to the interview, the researchers decided not to define concepts such as pain, discomfort or inability to perform duties, which are inherently socially constructed. As such, the same condition can trigger different levels of pain and discomfort between individuals or within the same individual at different points in time. One reason behind this choice is that explaining and defining such concepts to the respondents before the study would have failed to create a ‘common understanding’ of these constructs, because they remain inherently confined to the subjective sphere. Another reason is that such an attempt to quantify how respondents experience these constructs would have felt as an imposition of the researcher’s point of view and violated the emic perspective of gathering in-depth, subjective, socially constructed perceptions.

### Data analysis

Recordings and field notes of the in-depth interview series were transcribed verbatim in their original Bengali and Kannada languages. Bengali and Kannada transcripts were then translated into English. One Bengali and one Kannada speaking person were hired for this purpose. The first researcher double-checked all data for unclear passages and potential translation errors. Passages that raised doubts were cross-checked by the first and second author and, if applicable, further discussed with the translator.

Once the body of text deriving from the interviews had been cleaned up and processed, the data were analysed using the ‘thematic analysis’ method. This manual analysis adhered to the guidelines of Braun & Clarke [35]. Initially, the data were read through several times to ensure a thorough understanding. Patterns within the data were identified manually, coded and subsequently labelled according to their meaning. Codes sharing a common meaning were grouped under non-overlapping themes or categories. These themes were re-examined for further refinement by comparing them with the original statements in the participant’s accounts of their experience or perception of illness, their propensity to accept illness and their attitude towards reporting.

### Ethical considerations

In the absence of a formal committee to assess the research on ethical conformity, the study scrupulously followed the guidelines established by the National Committee for Ethics in Social Science Research in Health (NCESSRH)^3^,^4^ in order to conform to minimum ethical standards for doing research in the Indian context [36, 37]. Ethical approval for this study was provided by the Institute for Social and Economic Change (ISEC), Bangalore, India. All the respondents were told the purpose of the study, their part in the research, and the time and energy that they had to provide, as well as the possibility of pain, discomfort and stress that they might experience during the interview. Participation was voluntary. Verbal consent to tape-record the interviews was sought after explaining the purpose of the research to the participants and after assuring anonymity and confidentiality. The explanation specifically included the information that comments would not be attributed to a named individual without permission. The interviewers only tape-recorded interviews after receiving and noting down permission from the participants. In order to promote an atmosphere of trust, intimacy and informality, which was believed to create the necessary conditions for the respondents to feel at ease and respond openly and truthfully, written consent was not sought.

## Results

All the selected participants reported that they had suffered from at least one type of illness during the one-year period prior to the study. Overall, respondents showed an instant openness to talk about their illness; they expressed intense emotions or even talked about it for a long time with the researcher. However, they also revealed multiple situations (for example, pertaining to sexual and reproductive health or illnesses that are associated with social stigma) in which it was difficult for them to disclose their illnesses as openly to family, society and often professionals.

In general, the findings highlight that the decision to disclose illnesses as well as the choice of confidants is influenced by the complex interplay between gender, the nature of the illness, marital status and socioeconomic conditions. During the coding procedure and the surveying of the results, an attempt has been made to systematise the findings and to disentangle the main themes from the inherently much more complex nexus of causes. For grouping the themes, four main aspects of illness disclosure have been used which mirror our main research questions: 1. Choice of confidants; 2. Reasons to delay disclosure; 3. Reasons not to delay disclosure; 4. Reasons not to disclose. From these overarching topics, the following twelve themes emerged: healthcare professionals as the final recipients of illness disclosure; social norms and fear of future social sanctions driving the choice of confidants; bearable physical burden; negative prior experience of illness; coping with livelihood and everyday financial struggle; unbearable discomfort; therapeutic value of disclosure; fear of unfamiliar illnesses; previous negative outcomes of non-reporting or delay; withdrawal as a coping strategy; perceived threat to social image; and deteriorated atmosphere in slum environment. These themes are presented as motivating the choice of confidants, early illness disclosure, delayed illness disclosure or non-disclosure. However, because the themes per se are not connected to a specific category but are grouped in one category for the sake of systematisation, we number the twelve themes subsequently rather than in connection with any overarching topic. Table 3 summarises these themes, which portray the various reasons behind disclosure patterns among men and women.

**Table 3:** Overview of Themes and Subthemes behind Different Reasons forDisclosure Patterns among Men and Women

General category	Themes	Men	Women
Choice of confidants	*Healthcare professionals as the final recipients of illness disclosure*	Confidants: spouse and clinical doctors Reasons: share about chronic illness to ride out intense emotions and to receive treatment	Confidants: husbands and natal families are initial confidants for illnesses that lead to stigma, familial defamation and social penalty Reasons: feel that natal family members are more compassionate, attentive and considerate caregivers than in-laws
		Confidants: occasionally parents, close relatives and sometimes other people Reasons: people who are going through similar phase and can understand the distress	
			Confidants: in-laws are involved inmore common health problems Reasons: do not require emotional support
			Confidants: outside the family, informal healers are considered as the initial confidants Reasons: female-related illnesses and communicable diseases that primarily involve physical examination and that are complex to understand in the lay domain
			Confidants: clinical doctors are sought at the final or acute stage Reasons: mainly sought to cure illnesses
	*Social norms and fear of future social sanctions driving the choice of confidants*	Expressing illness is related to: time, place and person;illness considered as symbol of agony and distress may spread negative vibes during ceremonies and festivals, and can bring illfate to the people who participated Social sanctions:social penalty drives them to reveal only to spouses or natal families in order to avoid social exclusion, familial defamation and individual disgrace	Expressing illness isrelated to: time, place and person; talking about illness can bring ominous effect to the people who are related on auspicious occasions that are held at familial and social levels status, age and gender; prescribed norms drive single and young married couples to talk about common health problems with immediate and extended family Sexually related illnesses are open for discussion with informal healers (because they treat illness in trying to uphold cultural norms) but not with family members, non-kin and clinical professionals of the opposite sex, who can be deemed to share a liaison relationship
Reasons todelay disclosure	*Bearable physical burden*	Illnesses or pain that can be handled are considered too normal to report	Ignorance about the severity or effect of an illness results in leaving it unnoticed
	*Negative prior experiences of illness*	Job loss in the past prevents reporting the reappearance	Exclusion from any sociocultural participation at familial and community level creates an identity crisis for women
	*Coping with livelihood and everyday financial struggle*	Income crisis forces men to devote more time to the workplace and downplay the severity of the illness	Financial burden compels women to ignore illness as long as possible
Reasons not to delay disclosure	*Unbearable discomfort*	Unexplainable and unbearable internal pains are experienced as severe	–
	*Therapeutic value of disclosure*	–	By discussing illness, emotional, instrumental and informative support can be attained
	*Fear of unfamiliar illnesses*	Physical symptoms that cannot be linked to common health problems are cause for confusion, alarm and reporting	Skilled to remain calm and composed, and socially approved to report any unfamiliar symptoms immediately
	*Previous negative outcomes of non-reporting or delay*	Severe impairment to the body because of ignoring illness As a result, failure to carry out gender role as provider of livelihood	Severe impairment to the body because of ignoring illness As a result, failure to carry out gender role as caregiver
Reasons not to disclose	*Withdrawal as a coping strategy*	To retain normal balance of life as longas possible by self-coping with difficult feelings	–
	*Perceived threat to social image*	To secure one’s position in the family and society by safeguarding masculine ego	–
	*Deteriorated atmosphere in slum environment impeding disclosure*	Busy coping with adverse physical and mental conditions of the slum	Busy coping with adverse physical and mental conditions of the slum

### Choice of confidants

#### Theme 1 – Healthcare professionals as the final recipients of illness disclosure

The choice of the confidants to whom illness is firstly disclosed is markedly different between men and women, as well as being dependent on the nature of the illness. Women involve different people to disclose their illness at different levels. For instance, women initially said that they prefer and feel more comfortable to discuss their health problems with family members. By family members, they meant natal relations, particularly mother and sisters. Although in-laws and other affinal kin are occasionally involved, this choice depends on the nature of the illness. Common health problems are generally expressed to in-laws, but illnesses that lead to stigma, familial defamation, or negative and informal social sanctions are usually held back as long as they do not attract the attention of others. After natal kin, husbands are informed of illnesses the most often. Such reporting behaviour may find its roots in how families and society respond to and treat their illnesses. Women feel that natal family members are more compassionate, attentive and considerate than in-laws and their families. In this context, one woman said:

*Since childhood, I have been very weak and I am frequently seized by health problems... My mother is my power; she will always encourage me by making me feel special; whenever I am ill, she will not allow me to work, she will give me hot food, remain awake the whole night to check whether I am okay or not...My mother-in-law will not understand that. Instead, she blames my mother for giving them a physically unfit bride.*

Women in the study expressed that they experience severe anxiety and fear when it comes to female-related illnesses and communicable diseases which involve physical examination. Indian women are conditioned by multiple socially prescribed codes associated with sexuality surrounding marriage. For example, they are requested to maintain physical distance from men other than their husband, not to attract men by flaunting or exposing body parts and to protect their virginity before marriage. To keep things comfortable, women therefore report their health problems to informal healers, which marks the first step for them to report outside of the family. Informal healers are preferred to professionals, because the former generally reside in the neighbourhood and are well acquainted with all the households in the area, their health habits, practices and behaviours. As a result, respondents feel more at ease openly to discuss health-related problems at length. According to one woman:

*I talk at length [with the informal healer]. I tell him exactly how I feel about my illness; if it sometimes sounds funny, still he listens, comforts, encourages and helps me to come to terms with the trauma.*

Another reason for approaching informal healers in the study is women’s perception of such illnesses as complex conditions, which creates the need for them to confide in people who have knowledge about the illness and are better able to understand their mental stress. As one woman said:

*It’s okay to talk about your problems with family members, but they cannot always comfort you; there are certain female-related problems which are complex in nature and need to be understood by some knowledgeable person who can understand our emotions thoroughly.*

Reporting to formal healthcare professionals in case of health problems only happens when they become acute or severe. Women in the study expressed that professionals do not have the habit of allowing patients to share their views and distress about illnesses, or to give emotional and social support in order to cope with the illness. Rather, they conduct an immediate diagnosis and treatment whenever patients visit them. In this context, one woman recalls her experience:

*I went to him [the doctor], not with the intention to start treatment immediately but first to share my suffering and feel relieved. I expected him to comfort me by saying good and positive things and to give me the mental strength to deal with the illness, but he was focusing more on treatment than on listening to me and my feelings.*

This kind of distance which women perceive in relation to formal healthcare professionals influences their disclosure pattern, prompting disclosure to either family members or informal healers rather than to doctors.

By contrast, men prefer not to discuss general health problems with anyone, because they consider these problems to be too normal to discuss. In case of chronic or serious health problems, however, they report to their wives first and to the health professionals next. Parents, close relatives and possibly people who are going through a similar phase are occasionally informed about an illness in order to receive support when men are overwhelmed by negative feelings. Most men report involving their wives mainly to receive emotional support when they are going through a difficult time. Some men said that they disclose illness to their wives because they know that wives would not speak about a ‘*husband’s illness*’ to others, as this information will hurt both her and her husband’s self-image. As one man said:

*She feels that my illness is her illness and so she respects my emotions by not raising the issue with others*.

Outside of the family, men habitually hold back from informing friends, fellow employees and acquaintances. Many men found that when they tried to disclose their illnesses to these persons, their intense emotions were underestimated and their manhood was questioned.

Showing emotions and weakness was considered as uncommon for men, with many of them expressing that it is very essential in a group of men to show off one’s manhood even when they are experiencing immense internal turmoil. As one man said:

*Whenever all us men folk gather in the evening, we discuss downright everything from poverty, politics and problems in the family to the workplace; however, everyone is conscious not to discuss one’s health problem. No one reveals health problems, as this is the only place where men can show off their manhood and vigour.*

Unlike women, men find formal healthcare professionals to be quite efficient in providing comfort during stress. Many men in the study related that they can express their most intense emotions when they talk to doctors. This experience is linked to the way that they deal with formal healthcare professionals. Most of the men said that professionals react normally when men pour out their emotional turmoil related to illness. Some men also expressed the sensation during their communications with professionals that doctors find it quite natural and obvious that patients talk about their problems, irrespective of gender. In addition, formal healthcare professionals find the perception of manhood as related to health quite funny and irrational. As one man said:

*He [the doctor] started laughing and though it was a joke when I told him that I cannot express my feelings to friends in the same way as I did with him, because I will be labelled as a woman.*

#### Theme 2 –social norms and fear of future social sanctions driving the choice of confidants

The choice of confidants highlights a complex interplay of gender, status and the nature of the illness. For instance, many men and women who were interviewed expressed that young men and women, both married and unmarried, according to prescribed norms are allowed to talk about common health problems with immediate family (spouse, parents, children, uncles, grandparents, nephews, and so on), extended family (great-grandparents and other common ancestors) and family-in-law (parents-in-law and siblings-in-law) irrespective of age and gender. However, for specific illnesses such as female-related illnesses, sexually related illnesses and sexually transmitted diseases, discussion is not allowed between young and old or between genders when they share a liaison relationship^5^ such as brother and sister, father and daughter or father-in-law and daughter-in-law. As one woman said:

*It is very embarrassing and equally unmannered if you talk about female problems even when your father-in-law is in the other room*.

For other communicable or for chronic diseases, it is observed that no rules are prescribed. However, as these illnesses create penalties such as social exclusion, familial defamation and individual disgrace, participants said that it is they who create informal rules; for example, revealing it only to spouses or natal families in order to protect their honour. One man commented:

*Society has not told us to suppress serious health problems, but we sometimes have to do so in order to secure our and our families’ position in society*.

No rules have been prescribed for men in informing non-kin and professionals. However, as women’s relationships with professionals and non-kin (other than healers) of the opposite sex are considered to belong to liaison relationships, they are not allowed to talk freely about sexually oriented illnesses. As one woman said:

*People will look at you unfavourably if you frequently talk about sexual problems, even with doctors and other males.*

It is observed that women’s marital status and the nature of their illness also influence the way that illness is disclosed to the families. For instance, many unmarried women with abnormalities related to reproduction or imperfections of the body prefer an early disclosure of the illness. They consider marriage as essential to obtain both personal and financial protection. As it is their belief that society views them as vehicles to produce progeny, the chances of getting married are largely determined by how physically fit they are. Consequently, women and their family tend to ensure that any problem related to health is sorted first before it creates any obstacle to their marriage process. One woman talked about severe consequences which her sister had to face because of an initial failure to disclose her illness:

*She had been suffering from abnormal vaginal discharges since two years and had not informed anyone in the family, not even my mother. When my mother was finally told, she was shocked not so much by hearing about the illness but sensing that it may cause problems in her marriage...at that time, we were planning to get her married. My mother consulted a doctor who detected damage in her ‘baby pipe’ [fallopian tube].The doctor said that unless it is operated on, she cannot conceive. There was nothing we could do; the surgery would bring a huge expense that we cannot afford; everything was out of control. She remained unmarried, as we could not get her through [marriage] without telling the truth. Every alliance turned her down when they came to know the truth. There was no end to our problems. As time passed, we started to face new problems. As she is beautiful, she was naturally harassed often by some men from other localities. We even came to hear that there were men waiting for any opportunity to make a sexual move. It was becoming difficult for my parents to keep an eye on her every time and everywhere. As my sister started to feel unprotected, my father sent her to our village.*

In the case of married women, a few said that they sometimes reveal their problem to the family in-laws immediately as well, with the intention to gain their support and confidence. By talking to their mothers-in-law, women aim to show them that they are equally concerned about producing progeny. Indirectly, they also manage to secure their own marriage and financial security. One woman explained how this action worked positively for her:

*I took my mother-in-law into confidence by talking to her. I expressed my struggle with intense emotions and how I coped with them in battling the guilt of not being able to give them a grandchild. I also told her that I will do whatever is required to conceive and that’s a promise. By listening to my sorrows and pledge, her heart melted and she started to sympathise. She told my husband that I have no wrong intentions; in fact, I am trying hard to give him a child. She also told him to take good care of me in every aspect and to be by my side during this turmoil. Now, as my husband can’t disobey his mother, he is doing what he has been told. I am getting attention both mentally and financially. Of course, I am trying hard to sort the problem and give him a baby, but at least I can do so without the constant worry of being thrown out of the house and out of my husband’s life.*

It is equally essential for both men and women to take care of the way in which they relate their illness. Many participants expressed that time, place and person are crucial when it comes to sharing illness-related emotions. A woman said that they are not supposed to talk about illnesses on any auspicious occasions such as initiations, marriages, baby shower parties and religious ceremonies held at familial and social levels. As illness is considered a negative element, people believe that talking about it on joyous occasions and festivals will create an ominous effect. Another man expressed his belief as follows:

*Occasions and festivals are pious and holy moments where everybody is intent and bent on blessings, goodwill and affluence in life. As illness symbolises agony and sadness, talking about it on such occasions will damage the atmosphere and spread negative feelings which nobody wants.*

### Reasons to delay disclosure

Slum dwellers reportedly delay the disclosure of illness to family members, kin, non-kin and professionals because of various reasons. The main categories of reasons are bearable physical burden, negative prior experiences of illness and coping with the insecurity of the slum.

#### Theme 3– Bearable physical burden

Most men in the study looked primarily at biological aspects as determinants for reporting, such as the level of physical pain and their capacity to bear it. Physical pains that are minor and that can be handled are considered by men as belonging to general health problems and are therefore delayed in reporting. They psychologically made themselves resistant to general illnesses and consider these to be very normal in everyday life, as is reflected in one respondent’s attitude:

*Minor pains can be handled... they are not that serious; cold and fever are like frequent guests... they come and go; for these, I do not need to tell everyone immediately.*

As women in this case are far more sensitive, they are not seen to delay reporting. Although women do not intentionally delay reporting illness, however, a few of them said that they sometimes do so when they lack proper knowledge or information about the severity level or the effects of an illness. As one woman commented:

*I was not having my period for several months and I was ignoring it [considering it to be a normal phenomenon]. My mother told me that it was not normal, as it can create a problem while conceiving, and disclosed it to our* guruji *[informal healer].*

#### Theme 4 – Negative prior experiences of illness

For both men and women, expressing or reporting illness is related to their past experiences with the responses that they received from others. Illnesses related to unpleasant memories discourage slum dwellers from reporting when the same symptoms reappear. Men and women shared their experiences with unpleasant memories differently. Many men in the study stated that disclosing illness negatively affected their livelihood in the past. This situation meant that the frequent discussion of illness and the expression of feelings in the workplace signalled to others and to the authorities either the person’s lack of interest in his job and his desire to leave on the pretext of illness, or an attempt to raise his pay by triggering others’ sympathy in the name of illness. As a result, they had often been fired for displaying illness-related emotions and had had to bear a sudden financial crisis. One man described the difficulties that he faced when expressing illness in the workplace:

*It’s just that I was talking about my illness to one of my colleagues for two consecutive days. My boss noted this fact and misunderstood it as if I was doing so intentionally for him to hear. The third day, he dismissed me from the job, saying that he can understand my feelings of not being satisfied with the job and therefore inventing the excuse of illness.*

For women, unpleasant past experiences associated with reporting illness are more closely related to sociocultural context. Many women in the study found that too many negative discussions about their own illness with kin and non-kin result in exclusion from familial and social ceremonies. As one woman said:

*I used to tell all the negative things that I felt about my illness to my sister-in-law. Some months later, I discovered that she had not invited me to her baby shower ceremony. I was hurt. Later, I came to know from one of my distant relatives that she had been saddened and that she feared a bad omen. Therefore, she did not want me to be there during that auspicious ceremony. I know all that happened because of my talking too much about bad things.*

#### Theme 5 – Coping with livelihood and everyday financial struggle

Both men and women commonly acknowledged that their livelihood and the everyday financial struggle which they face prevent them from promptly disclosing their illness to anyone. Instead of focusing on health and illness, the slum dwellers feel compelled to concentrate on securing the basic necessities of life. For instance, the struggle to retain their job plays a crucial part. Most slum dwellers work in the informal sector under the constant threat of losing their jobs. It is important for them to secure their job by making an extra effort and showing their commitment to the job, by meeting the daily or weekly targets and by remaining present onsite for long hours. Many men who were interviewed expressed that they do not consider illness important enough to be shared and discussed, as it will bring no immediate harm to their livelihood. Rather, it is their bread and butter which are primarily affected if they do not talk with others about the job market situation or think about better chances in employment. One man said:

*My health will not take away my bread and butter if I do not think or talk about it for five days. But if I do not show my commitment to work and do not take it seriously for even one day, I have to think for the other five days what my family will eat.*

Consequently, some men mentioned their attempts to divert their mind from thinking about illness or burdening it too much in order to secure their wages. As one man said:

*If you talk about illness, this means you are thinking about it constantly, and thinking about it means you are actually not well. The next day, you take a leave from your work feeling very sick; and your one-day wage is gone. The moment you stop talking about it, you will find life is normal...it’s all about your mind.*

Closely related to this topic are financial struggles, which involve a lack of or a meagre family income, depletion or lack of savings, unemployment or underemployment, excessive debt and uncertainty about the future flow of income. All of these factors force slum dwellers to consider health as a secondary aspect. Some women said that they delay the disclosure of health problems to professionals because they find that the professionals are expensive. They fear that going to professionals early on will bring crises after treatment, such as selling or mortgaging property because of debt. On this topic, one woman commented:

*Forget about sharing things with him [the doctor]. Once you go there, he will immediately start his expensive treatment and you have to start selling everything for the treatment. The later you go, the better. At least then, the impact of the crisis will be less.*

### Reasons not to delay disclosure

This theme illustrates the reasons that prompt the participants of the study to express or report illness. In many cases, the intention behind informing others about illnesses in time is related to finding solutions, coping with the distress or preventing potential collateral damage. The themes that emerge are unbearable discomfort, therapeutic value of disclosure, fear of unfamiliar illnesses and previous negative outcomes of non-reporting or delay.

#### Theme 6 – Unbearable discomfort

Men report disclosure when the pain becomes unbearable or unusual, as reflected in one man’s comment:

*I have had enough of this leg pain and cannot bear it anymore. I reported itto a doctor immediately.*

Some men noted that it is difficult for them to assess the intensity of illnesses which occur internally, due to a lack of knowledge related to human anatomy or body functioning. Any strange internal pain or discomfort that happens, even for the first time, is therefore experienced and labelled by them as severe and reported without delay. As one man said:

*One day, I vomited three to four times. It happened to me for the first time. I felt something very wrong was going on inside. I got so scared that I immediately talked about it to one of the local doctors.*

#### Theme 7 – Therapeutic value of disclosure

Women are found to be very sensitive to illness or bodily discomforts and show more willingness to express symptoms of distress to others for psychological relief as compared to men. Sometimes, expressing distress rather had to do with the complexity of the female reproductive system and represented a coping mechanism and a deliberate act of self-encouragement. Most women consider female-related health problems as unavoidable because of the complexity of the reproductive system. Such belief has emerged from their socialisation process, as they have been told and made to believe since childhood that their biological composition is responsible for their morbidity and even puts them at risk of death.

As a result, women developed the coping strategy of reassuring themselves by continuously expressing their distress to others. As one woman said, reporting to others actually helps women to comfort themselves that everything will be alright and that reproduction-related morbidity will not necessarily lead to death. The same woman mentioned the following:

*We tell our distress only to hear from others about women whom they know, who led a healthy life and died due to old age. By listening to all these accounts, we actually try to create hope in ourselves that we can also live in the same way and die due to old age rather than from female-related problems.*

Moreover, they share their health problems with each other to find out various possible solutions to similar experiences that they have had in relation to illness. Such sharing of experiences raises increased interest among the women who face challenges. One woman described how she benefits from the illness-related talks with her friends:

*Every evening, we sit and chat about our daily life experiences; talking about health is one of our favourite topics. We have come to know many unknown things from each other, and we share various techniques and methods to protect and prevent illnesses from occurring. This information seriously helps...at least, I am confident that I can come up with whatever is needed to protect me and my family*.

Many women find that a failure to express or disclose one’s illness even for a single day means allowing the illness to breed inside the body. They further feel that this approach will yield nothing positive but will instead bring unhappiness in life, as well as weakening one’s immune system and strength to survive. As explained by one woman:

*The sooner you pay attention to health problems, the better you feel. These things are not meant to be hidden; at least, they should be shared with family and friends the day you sense them. Otherwise, the distress will eat you up slowly, making you more miserable and lifeless.*

#### Theme 8 – Fear of unfamiliar illnesses

One of the major concerns raised by men and women in the study relates to their unfamiliarity with symptoms or their lack of knowledge with which to assess the severity of the illness. Symptoms that do not show similarities with common health problems such as heart conditions, lung problems or abdominal pains are difficult to interpret and therefore cause preoccupation. Such symptoms include the presence of any kind of blot or patch without pain, any unusual disfigurement or any prolonged unexplained illness.

Many men realise that they very easily get anxious about such unknown symptoms and start creating a commotion within the family. As one man said:

*I got very scared... the whole area around my genital parts was full of several abscesses...I only sensed that something terrible had happened to me and immediately informed my wife*.

Some men said that they get puzzled and therefore feel the strong sense to report it to the professionals. One man who was suffering from cyanosis recalled his first reaction:

*I was too confused to even think about it, I have never seen or heard such strange things happening to others. It was awful to see the colour of your skin changing. Without wasting any more time, I immediately asked a doctor for help.*

Such non-delay in reporting is due to a lack of knowledge about the nature and severity of these unfamiliar symptoms whose effect cannot be predicted.

A number of women in this study also experienced unfamiliar symptoms. They immediately disclosed them to the family but remained composed while dealing with the situation, as they are mentally prepared to accept and adjust to any kind of illness. As one woman said:

*These things [unknown illnesses] are very dangerous; you never know how it’s going to spill over. In this situation, you need to be very cool and calm in order to act wisely. Throwing tantrums over it will only make it worse*.

#### Theme 9 – Previous negative outcomes of non-reporting or delay

As already discussed in an earlier section, the previous experiences of the participants relating to the expression of illness can become a major cause of their delayed reporting. However, this factor is an equally important reason for the timely reporting of illnesses. Such reporting patterns vary with the situation, though. In this context, for instance, the immediate reporting of illness is linked with unpleasant memories of the past where ignoring illness had made the participants face intolerable pain or serious damage to the body. Many men and women in the study related the unforeseen consequences that they had to face because of delaying disclosure or not reporting illness. One man commented:

*Once, I fell off a truck. I did not find it that important to discuss with everyone. Within a month, I realised that I could not sit or stand straight. After consulting with the doctor, we discovered an injury in the spinal cord that had actually occurred during the accident. I was out of work for almost seven months and there was no earning in my house*.

One woman said:

*My right hand became paralysed and remained invalid for several years because of disregard. I felt that I was becoming a burden to the family-in-law, as I was of no help [in doing household chores]. They were angry that I had not at least informed them about my illness. They blamed me solely for my situation.*

As a result, the participants are scared to take a second risk when they experience similar symptoms relating to the mishap and therefore report the problem immediately to the family or the professionals.

### Reasons not to disclose

It seems that men and women in the study sometimes deliberately withdraw themselves both emotionally and verbally from family and others. In this section, we are concerned rather with the explicit notion of avoiding to express feelings in relation to illness.

#### Theme 10 – Withdrawal as a coping strategy

Most men noted that they try to avoid highlighting problems by deliberately downplaying their impact or severity. The function of this behaviour could be interpreted as the avoidance of difficult feelings by self-coping with illness and looking for a way towards a normal life. One man described his strategy as finding ways in daily life to prevent illness from entering the mind:

*I do not let any illness hover in my mind...I talk to others about the usual things rather than about illness, I play with children, do light work at home, play cards with friends in the evening and go to the local temple. These things help me to distract myself rather than getting preoccupied with illness.*

Some men feel that telling others is equivalent to allowing emotions to grow, which further deteriorates their mental state. In this quest of finding a normal equilibrium in daily life as soon as possible, they therefore refrain from reporting illnesses to others. As one man said:

*People will not let you forget your illness if you share it with them. They will constantly make you feel ill by asking every time you see them how you are feeling and what steps you have taken to deal with it. It’s a struggle with yourself and your will, so I find that it’s easier to make yourself feel normal by not letting others know about your illnesses.*

#### Theme 11 – Perceived threat to social image

Although men do not have to bear social consequences to the same extent as women, they nevertheless remain under the pressure of gender-related societal perceptions where they are expected to be strong, tough and resistant to illness. As a result, men expressed that they find illness to reduce their status in the male hierarchy. For a man, displaying toughness represents a badge that they always have to wear, lest they lose their value and identity as a man in society. The more they bear pain and suffering, the more their degree of manliness increases. Many men consider masculinity as a symbol of strength, while talking about illness is seen as a ‘feminine’ thing. One man said:

*We cannot show our health problems, you know...it’s a female thing to show weakness... we will become the laughing stock of our friends.*

Another reason why men do not reveal their health problems in the family is linked with a shift in positions of power with women. As one man said:

*I was bedridden for months and Razala [his wife] started taking me for granted in every aspect...I felt powerless. It’s really very difficult to accept that suffering from some health problem makes you invalid; your family, who once used to respect and listen to you, starts taking you for granted. I don’t want this to happen again, so it is better to keep the illness within myself.*

One man described the erectile dysfunction that he developed for some months as a consequence of ignoring his diabetes symptoms:

*My doctor told me that it happened because of not checking my diabetes. Actually, I did not find it [the diabetes] that important to talk about...It was a blow to my manhood. I felt like committing suicide.*

#### Theme 12 – Deteriorated atmosphere in slum environment

The slum dwellers occupy marginal positions in the sociocultural system of the community, inhabiting an unfit environment. In this respect, some male and female participants expressed that they struggled to cope with many slum-related practical issues such as environmental decay, poor infrastructural facilities, displacement, poverty and crime. All of these aspects severely undermined their sense of safety and security. Many of them described their daily struggle in relation to some of these issues. Prior to focusing on any health-related activities such as identifying, disclosing and treating illnesses, they believe that faulty basic infrastructure should be addressed such as improper toilet facilities, improper shelter, open drains or sewages and that basic needs should be satisfied including proper sleep, clothes and food. The feeling of insecurity when these basic needs are not met can overpower their attitude towards sharing illness. As one woman said:

*If there are no proper water and toilet facilities, it is obvious that we will get ill...so there is no question of talking about illness and wasting time until and unless these problems get sorted.*

Slum dwellers deal with life-threatening situation and sexual violence. As they have had to face the challenge of frequent displacement, they often become victim of theft, murder, molestation and rape. For women, securing protection during displacement and finding their way among various uncertainties hardly provide any room for illness. One man talked about his struggles during displacement and his constant fear, which do not leave any space to talk about illness:

*When we all were thrown out from our previous location... We all came to a land that was already littered with waste and that was filthy. I somehow made a small, fragile tent to ensure my family’s security; I had two adult daughters during that time and was worried about their protection. I used to spend sleepless nights keeping a watch on my daughters’ safety. My son used to study in a nearby school free of cost, but he had to stop that too because of the distance. Even now, we live every moment in constant fear of being thrown out from this land as well. Now I am old and cannot struggle as before. After listening to all these things, do you think that we have the mental condition to sit and talk about illness? Whenever I suffer from any illness, I find a way out for myself. I do not have the habit of discussing it. Everyone here is busy fixing their own problems. So it’s as though I neither have the patience to discuss illness, nor do people here have the patience to listen.*

## Discussion

In light of the emic perspective used in this study, the novelty of its work resides in unravelling the patterns of illness disclosure within the specific setting under examination, to wit Indian urban slums. Low-income, resource-constrained settings are characterised by a high level of idiosyncrasy and specificity, which impedes any generalisation of findings to other contexts or a transfer of evidence collected in other contexts to the one under investigation. As a consequence, findings and patterns found in other settings – even in the Indian context – cannot be used as a vantage point to understand illness disclosure behaviour in Indian urban slums.

Our findings painted an expansive portrait of the patterns of illness disclosure. Although the factors for men and women are similar in the studied slums, men and women often related different reasons underpinning their disclosure pattern. Our findings also indicate that the choice of illness disclosure is basically a coping mechanism adopted by participants to deal with any adverse situations linked with illness, which is related to whether they suspect the illness to constitute danger both for themselves and for the community. The decision to delay, not to delay or to avoid illness disclosure is not determined solely by the recognition of illness, but largely depends on the interplay between their agency, the consequences of disclosure and the sociocultural environment [38].

The present study indicates that women are more active than men in expressing their illness to others. Although they involve several people as confidants, they have reservations in particular about the way that they disclose illnesses. This finding is consistent with existing literature which has shown that women are more frequent disclosers than men [39–41]. It is observed that natal families and husbands are the major resources of moral and social support as compared to other kin and non-kin. The literature identifies family as the initial and most important source of support for patients, since the highest level of social support is received from the family [42–45]. However, the quality of social support is equally important to women. For this reason, they mobilise the support system of informal healers for both social and informational support in complex problems of reproductive health. The choice to mobilise professional support is related to women’s need for specific medical interactions and actions rather than just lay discussants.

Most of the time, men avoid expressing their emotions regarding illness to others, except in the case of fatal illness. They have a very limited network of support and mostly rely on their partners and on professionals. When men experience any vulnerable situation with illness, in order to receive special attention they sometimes open up to relatives and friends who also went through similar situations. Partners are approached to receive emotional support and to keep illness secretive, while professionals are sought mainly to gain pre-emptive sympathy without harming the dominant status of their hegemonic masculinity. Earlier studies have also reported that men and women differ in their sources of support [46, 47]. Men basically rely on their partners as primary sources of support, whereas women draw support from a broader social support network [48].

It is important to see that the participants’ cautious disclosure of illness is also articulated within the culturally prescribed norms. These norms are similarly reported in other studies, revealing that the decision to disclose is influenced by cultural orientation [49, 50]. The desire to share illness is controlled by the state of affairs, status, age, gender and the type of illness. Stigma and social exclusion specifically attached to some illnesses, such as those related to sexual and reproductive health, appear to influence disclosure behaviour heavily in terms of disclosure delay and choice of confidants. As a consequence, illness disclosure behaviour – particularly that of female participants – cannot be viewed in isolation and should be understood within a complex nexus of determinants related to gender, type of illness, type of confidants, and socioeconomic and sociocultural conditions. Though men are more socially and psychologically adaptive in remaining secretive about illness, women cannot always express illness either and sometimes have to hold their feelings at bay in order to minimise the impact of unsupportive social interactions. Negative reactions that result in adverse social support are in fact a strong predictor of non-disclosure, as seen in the findings of another study [51]. Culturally prescribed norms have also created the preconditions for selective confidants of selective illnesses in both men and women. This finding confirms previous studies which have shown that family cultural norms govern the rules regarding appropriate expressions or acceptance of illness [50, 52]. As women utilise more support during crises, common culture accordingly promoted women’s habit to involve more confidants overall. In turn, the impact of culture among men is undermined as they are psychologically bound to talk less about illness [53]. However, in the case of fatal illnesses, the cultural constraints that discourage participants from full disclosure so as to avoid the burden of isolation, social sanction, and the rejection of both oneself and one’s family grow weaker and at times overlap with the social norms.

In our findings, we observed that the choice of disclosure is a complex decision based on the potential risks and benefits of disclosing, which are in turn multifaceted and which rely on factors such as gender, type of illness, marital status, timing, confidants, and so on. In the literature, difficulty in choosing to disclose has been documented [54–56]. Men’s approach to delaying or avoiding the disclosure of illness may be generated by self-perceived images of masculine health-related beliefs and practices, as well as by the fear of not being able to meet the gender role expectations both in the family and at work, framed as a self-focused coping strategy for battling with the insecurity of being a slum resident. By contrast, women’s delay in disclosing illness is linked to the biological, social and psychological challenges unique to their disadvantaged social position. Again, while men share their illness to others without delay mainly to prevent or cope with biological problems, women promptly report illness for security or emotional and instrumental support when they face severe and unknown biological challenges, unforeseen crises and a loss of security similar to what they have previously experienced. Regardless, the slum environment plays a pivotal role in shaping the attitude of the slum dwellers towards disclosing illness. Structural phenomena unique to the slum community (disintegrated life, poverty, inequality, crime, fragile physical infrastructure) have an overpowering effect on their health-seeking attitude. As their struggle of being in a slum community leaves no space for health concerns, the need to report illness is hardly recognised. This construction, evoking a complex nexus of needs, desires and powers behind health-seeking behaviour, conforms very well to Maslow’s theory of motivation based on the ‘hierarchy of needs’ [57]. This theory explains that higher-level needs such as health, family, relationships, security of environment and employment are dependent on the fulfilment of lower-level innate or basic needs such as food, shelter and sleep. The slum dwellers in this study confirm that health is perceived as a higher-order need, which can be addressed only once the more basic needs have been met.

The strength of the study lies in its large number of respondents and its multi-dimensional approach, in which the perspectives of both male and female respondents as well as the context of disclosure are taken into account. Its results have implications for the nature of disclosure among slum dwellers. These insights can inform future interventions, in which the supply of health resources can be shaped so as to meet slum dwellers’ needs. The study also suffers from a number of limitations. First, both men and women were interviewed by the main researcher, a woman. While this fact favoured the openness of female respondents, who are traditionally more reserved, it could have introduced a bias in gathering male perspectives. Second, the perspectives of partners, kin and non-kin linked to the individual participants were excluded. Although we found the family to be a source of disclosure and support, we did not examine their subjective views about the participant’s disclosing behaviour. Third, the incorporation of the individual’s perception of illnesses as suffered more than one year before the study period could have revealed more patterns of disclosure which were not reported by participants reviewed here. However, it might also have introduced a significant bias in recalling experiences that are further removed in time. Fourth and finally, because the participants were left free to interpret or use self-constructed concepts and meanings of illness, pain, discomfort, inability to perform duties and any other illness-related aspects, such notions are likely to differ in future and across people. In line with a social-constructivist emic perspective, these findings cannot be generalised to other social groups or to the same social group over time.

## Conclusion

The findings of this study demonstrate that the relationship between the act of disclosing and the characteristics of illnesses is dependent on the typical sociocultural settings where the behaviour occurs. Men tend to delay disclosure or to prefer non-disclosure and have a very limited number of confidants. The disclosure of illnesses is delayed or avoided because physical pain in some occasions can be kept under control, to protect one’s job and income, to retain a normal life as long as possible by undervaluing illness and to secure one’s position in the family or society by safeguarding masculine ego. Prompt reporting is mainly associated with prevention and coping with biological challenges that people experienced before. Women involve several confidants at various stages of illness, which attitude can be linked with culturally mediated support systems. Reasons for delay or non-disclosure are a lack of information about the nature of the illness, avoiding stigmatisation or emotional loss because of social exclusion and identity crises, financial crises pertaining to healthcare expenses and struggling with the adverse situation of the slum. Prompt disclosure of illnesses is related to receiving therapeutic experiences in sharing illnesses, gaining confidence to fight unknown physical complications, preventing the recurrence of negative consequences due to delay or non-reporting, and avoiding future social sanctions. The study may indicate a pathway to policymakers and proves that disclosure comprises an important component of health-seeking behaviour prior to seeking treatment. For a successful use of healthcare resources and the improvement of healthcare utilisation by slum dwellers, disclosure patterns therefore need to be addressed within the local sociocultural context where slum dwellers reside. This approach can be realised by encouraging the slum dwellers to share their feelings about illness more openly through decreasing their sense of marginalisation, removing the inhibition to disclose to certain people and increasing professionals’ awareness of unsupportive social interactions.

## Abbreviations

EM: Explanatory Model of illness
ISEC: Institute for Social and Economic Change
NCESSRH: National Committee for Ethics in Social Science Research in Health

## Footnotes

1 The present paper is part of a broader qualitative study that examines health-seeking behaviour in terms of the health beliefs, health practices, reporting behaviour and treatment-seeking behaviour of urban slum dwellers in four different slums; two each selected from two Indian cities.

2 Individuals in the category of non-disclosure who experienced illness in the one year prior to the study chose not to disclose their illness to anyone at the moment when the problem occurred, but they did report the illness episode to the researcher.

3 See http://www.cehat.org/go/uploads/EthicalGuidelines/ethicalguidelines.pdf.

4 See http://www.anusandhantrust.org/centres/Ethics_in_health_research_-_A_social_science_perspective.pdf.

5 Considered as a secretive or adulterous sexual relationship.
